# Case of Inflammation of the Brain and Spinal Marrow

**Published:** 1839-06-01

**Authors:** 

**Affiliations:** Resident Physician


### MEDICAL EXAMINER.

DEVOTED TO MEDICINE, SURGERY, AND THE COLLATERAL SCIENCES.

No. 22.] PHILADELPHIA, SATURDAY, JUNE 1, 1839. [Vol. II.

*Case of Inflammation of the Brain and Spinal Marrow. Philadelphia Hospital. Clinique of* Dr. Gerhard.

(Reported by Dr. Cottman, Resident Physician.)

John G., æt. 27. Patient entered Men's Lunatic Asylum, May 14th, 1839, in a state of great excitement. He is a large, muscular man, well formed; expression of countenance fixed; eyes rolled upwards; pupils very much dilated; conjunctiva injected; tongue coated and dry; breathing laboured; skin rather above natural temperature; when asked a question, gives some vague and unmeaning answer; can get very little of the anterior history of the patient from his answers; says, however, that he was born in Scotland, has been in this country some six or seven years, during that time has worked at various trades; at one time a sailor, at another a fisherman; last summer was much exposed to the sun on the water, and to that he attributes the loss of sight of both eyes. There is complete amaurosis in both eyes; sense of hearing very acute; thirst very great; talks constantly in broken and unconnected sentences; has evidently been a very intemperate man.

℞. Tr. Valerian. ʒvi.

Spt. Eth. Sulph. Comp. ʒiv.

Pulv. Gum Camph. ʒss.

Mist. Assafœt. gr. ft. ℥vi.

Sig. Sumat. sem. unc. secund. quâque hor.

Apply a blister to the back of the neck, seven inches by five. Cold applications to the head.

[The mixture of valerian and assafœtida was a temporary prescription, which had been given at the entrance of the patient, to be discontinued if the case should become more decided.]

Saw the patient in the evening; found him in a state of stupor; with much difficulty aroused, even when spoken to in a loud tone of voice; succeeded in arousing him once, but immediately sunk into the same state of stupor; head very hot; extremities cold; tongue harsh and dry.

*Treatment.*—Sixty leeches were ordered to be applied to the temples and behind the ears; cold applications to the head; mustard cataplasms to extremities.

*May* 15*th.*—10 o'clock, A. M. Patient slept two hours; an exacerbation of all the symptoms; fever very high; skin hot and dry; pulse 120, corded; an increase of cerebral symptoms; talks much, and very incoherent; constant nictation; pupils dilated, the right more than the left; tongue coated with a white fur, very dry; craves ice constantly; breathing laborious; bowels have been in a constipated state for three days.

*Treatment.*—Venesection as the strength of the patient will bear.

℞. Hydrarg. Chlorid. Mit. gr. x.
Pulv. Scammon.
Pulv. Gambog. aa gr. xv.
M. Sig. Statim. Sumend.
℞. Ol. Terebinth. ℥i.
Muc. Lini. ℥iv.
Sig. adhibeatur formâ enematis.

Ice to head; mustard cataplasms to extremities.

4 o'clock, P. M. Medicine has not operated; patient still incoherent, talks constantly; slight abatement of febrile symptoms; thirst very great; face bedewed with perspiration; skin over the whole body, moist. Repetatur enema. Continue sinapisms.

9 o'clock, P. M. Patient in a state of complete stupor; cannot be aroused, not even when spoken to in a loud voice; eyes dull, pupils dilated; breathing laborious; moans constantly; skin natural temperature; pulse 130, when first examined; sank to 90 in the course of twenty minutes, and rose again to 120 in fifteen minutes; bowels were open very freely after the second injection. Repeat the leeches to temples and behind the ears; ice to head; mustard cataplasms to extremities.

Died at 3 o'clock, A. M., May 16th.

*Autopsy thirty-two hours after death.*—Exterior well formed, presenting nothing remarkable.

*Head.*—Liquid blood in sinus; large quantity on dura mater; veins at summit of brain distended with dark blood; small vessels at summit of brain much injected, particularly on right hemisphere, where the injection is very abundant; membranes opaque; arachnoid very dry, no serum is found beneath; the pia mater is readily detached; the convolutions of the brain are rather pale, and somewhat flattened; on left side membranes equally pale, but convolutions not so much flattened. The cortical substance of the right hemisphere is of a light pink colour, and generally firm; medullary substance is moderately injected, and firm, except at the posterior third of the cerebrum, where in a space of one and a half inches it has a yellow, cream-like colour, dotted with spots of blood, which render it, in spots, of a lilac tint. The softening extends to the cortical substance at the posterior margin of the brain. On opening the right ventricle, well-formed greenish pus issued out with rapidity, this had evidently been contained within the ventricle, amounting, in the whole, to one ounce. The membrane lining the ventricle was injected; at the posterior extremity of the ventricle is found a deposit of lymph, covered by the purulent liquid. The left ventricle contains an ounce of transparent serum; its walls are not injected.

The septum lucidum is softened, and slightly injected; fornix softened. The membrane covering the septum lucidum, on right side, is evidently thickened. A cluster of hydatids is found on the posterior part of the plexus choroides in the left ventricle; in the right ventricle it is infiltrated with pus, and bound down by adhesions; the thalami and corpora striata are firm, and moderately injected.

*Base of Brain.*—Minute injection of the vessels at summit of the brain, is observed also at the base, and is nearly equal on both sides. The membranes covering the lower surface of the left lobe of the cerebellum are entirely opaque, from a deposit of green puriform lymph beneath them; on the right lobe the same matter was perceived, but in less quantity. The lymph is also effused around the optic nerves, and behind them, but does not extend to the fissure of Sylvius, and presents no tubercular granulations. At the anterior lobe of the cerebrum, the injection is much more distinct on right than on left side; the upper surface of the cerebellum is scarcely injected; its substance is perfectly firm and of the usual colour. The lining membrane of the fourth ventricle is brightly injected, covered with a delicate layer of lymph, opaque.

*Spinal marrow.*—Dura mater much injected within the cavity of the arachnoid membrane; extending along its whole length is seen a greenish coloured pus, amounting to one or two ounces, evidently contained within the arachnoid proper. That portion of this membrane which lines the dura mater, is covered with a coating of lymph, but is less injected than that covering the medulla. A purulent liquid is contained beneath the arachnoid. The medulla is softened, irregularly; in its dorsal portion the softening extends to the whole posterior columns, and in part to the anterior; the softened portion has a colour similar to that of the brain, but is less intensely red.

*Heart.*—Pericardium contains a few drachms of serum; valves flexible; volume and thickness of the heart of the usual standard.

*Lungs* engorged with serum; slightly emphysematous along the margin, offering a few points of melanosis.

*Liver*, natural size, not fatty, engorged with blood, bile thick, dark.

*Stomach* not distended; internal membrane mammillated, thinned in patches near the small curvature, of a dull slate colour. Glands of Peyer perfectly natural, and throughout its whole extent the small intestine is pale and firm. Large intestine was examined in several places, perfectly firm, and contained thin greenish fæces.

*Spleen* very small, firm.

*Kidneys* firm, exhaling a strong odour of turpentine.

This case is necessarily deficient as regards the anterior history of the patient, who entered in a state of delirium; we were unable to obtain from his friends any information at his entrance. The disease was represented to be delirium tremens; and some of the symptoms were so similar to those of the second stage of this affection, that not a little doubt remained as to the true nature of the disease. It was distinguished, however, by the complete incoherence, and the absence of the great contraction of the pupils, and of sweats. In the latter stages of delirium tremens, it is usual to meet with the complete incoherence of ideas; but we do not often observe this symptom, unless the sweating is at the same time profuse.

The incoherence gradually passed into stupor as the disease advanced, but there was no distension of the face, and little or no rigidity. The limbs may have been slightly rigid, but at least they were not so to a sufficient degree to excite attention. There was no convulsion, or even subsultus, observed at any time.

The inflammation was, therefore, almost completely latent; at least the symptoms were extremely obscure. The nature of the disease was recognised before death, but not its extent. It was certainly not at all probable that the affection had reached the stage at which life terminated. Probably much of the obscurity would have been removed if the patient had been seen at an earlier period; but, we speak of the case as it appeared under the actual circumstances, and as adding another instance to the mass of facts already existing which show that meningitis is often a very obscure affection, and one that may readily be confounded with several others of a different nature. These are delirium tremens, insanity of the true kind, some forms of hysteria, and typhoid fever.

We are led to infer that the purulent effusion had already commenced previously to the entrance of the patient, and, of course, all treatment was unavailing. Perhaps a different result would have followed, if the patient had been treated at the commencement of the affection.

The reader will perceive that the inflammation was unusually extended; it not only included the pia mater, but a considerable portion of the cerebral substance, and the arachnoid proper. Purulent lymph and serum were contained in the cavity of the latter membrane, both in the brain and spinal marrow. This great extent of the disease on both hemispheres, rendered the case more difficult of recognition, and destroyed the contrast which usually exists between the symptoms of the sound and of the diseased half of the central nervous system.

